# Electromagnetic Frequency Pollution in Malawi: A Case of Electric Field and Magnetic Flux Density Pollution in Southern Africa

**DOI:** 10.3390/ijerph20054413

**Published:** 2023-03-01

**Authors:** Fabiano Gibson Daud Thulu, David Tembo, Rowland Nyirongo, Patrick Joseph Cardinal Mzaza, Allan Kamfosi, Upile Chitete Mawenda

**Affiliations:** 1 Physics and Biochemical Sciences Department, Malawi University of Business and Applied Science, Chichiri, Blantyre 312225, Malawi; 2 Department of Mathematical Sciences, Malawi University of Business and Applied Science, Chichiri, Blantyre 312225, Malawi; 3 Department of Physics, University of Malawi, Zomba 305218, Malawi; 4 Kamuzu Central Hospital, PAM Department, Lilongwe 207232, Malawi

**Keywords:** electric field strength, electromagnetic measurements, electromagnetic pollution, magnetic flux density, environment, health effects

## Abstract

In this study, electric field and magnetic flux density pollution levels were measured in southern Africa, a case of Blantyre City in Malawi, between 2020 and 2021. Sixty short-term measurements were performed using Trifield Electro Magnetic Frequency meter model TF2 in 30 different locations. Five high-population-dense sampling points were selected from school campuses, hospitals, industrial areas, markets, residential areas, and within the commercial and business center (CBC) of Blantyre. Electric field and magnetic flux density pollution monitoring was conducted between 10:00–12:00 h and 17:00–19:00 h for short-range analysis. Short-range results show that the maximum measured electric field pollution were 249.24 mV/m and 207.85 mV/m between 10:00–12:00 and 17:00–19:00 respectively, which are below the public limits of 4200.00 mV/m for public exposure. Similarly, maximum short-range results for magnetic flux density were 0.073 G and 0.057 G between 10:00–12:00 and 17:00–19:00 respectively which are below the public limits of 2 G for public exposure. Both measured electric and magnetic flux density were compared with the International Commission on Non-Ionizing Radiation Protection (ICNIRP), World health organization (WHO), and Institute of electrical and electronics engineers (IEEE). It was determined that all measured values for both electric and magnetic flux density were smaller than set limits for non-ionizing radiation for both public and occupation health. More importantly, these background measurements establish a baseline for future changes to be compared against public safety.

## 1. Introduction

As technology is growing in Africa, the exposure and dose of electromagnetic frequency (EMF) to people will continue to rise. EMF pollution is currently one of the foremost environmental qualities and a problem associated with technology in Africa. Implications from electromagnetic radiation pollution exposure are in both urban and rural areas which influence human health before conception, during pregnancy, childhood, adolescence, and up to adult life [1]. Several anthropogenic activities have resulted in the release of EMF waves, mostly into the urban environment [2]. Technogenic sources of EMF pollution in populated places include mobile communication, electric base stations, wireless and cable Internet, radio equipment, high energy voltage transmission lines, mobile communication, research and medical devices, and others [3]. Exposure assessment studies show that people are mostly exposed to EMF from electrical appliances and tools, power lines, and from power building supply. People who work near transformers, electrical closets, circuit boxes, or other high-current electric equipment may have 100 V/m electric field exposure. In ordinary work places such as offices, electric field levels are similar to those found at home; however, these levels may increase dramatically near certain types of equipment [4]. All these EMF emitters are available in Malawi.

Anthropogenic activities significantly exceed natural background sources, therefore, becoming a dangerous contributing factor to the pollution of the general public and the environment. Since each electric transmission line at any base station is an EMF source, it is difficult to fully eliminate the exposure of EMF source in our living environment [5]. The science of measuring, evaluating, and monitoring the exposure levels of EMF pollution while also qualifying the deterministic effects of such frequencies on human health has become more crucial to the public. Hence, this topic should comply as ongoing research to be conducted in Malawi. The levels of electric and magnetic exposure levels in Malawi are not known. This work and the upcoming continuous monitoring will provide information on the trends of both electric field and magnetic flux density pollution levels in Malawi. More importantly, these background measurements establish a baseline for future changes to be compared against. Research into electromagnetic pollution, shielding measures, and mitigation of biological effects is also vital to the optimization of electric transmission systems in Africa.

There are international guidelines on limits of exposure of humans to EMF pollution. Malawi has no established guidelines on this subject yet, as such, it adopted the international guidelines [6]. It is required that each country has to set its guidelines on EMF exposure limits [7]. It is recommended by the International Commission on Non-Ionizing Radiation Protection (ICNIRP), which is recognized by the World Health Organization (WHO) and the Institute of Electrical and Electronics Engineers (IEEE) that these exposure limits be based on a 24 h duration exposure [8].

For a person who is standing directly under a high voltage transmission line, he or she may feel a mild shock when touching something that conducts electricity. These sensations are caused by strong electric fields from high voltage electricity in the lines. They occur only in close range vicinity because electric fields highly reduce as a function of distance. Electric fields might be shielded and rapidly weakened by buildings, trees, and other objects that conduct electricity [9].

In Malawi, the wireless systems and base stations operate with frequencies below 500 GHz, which falls in the non-ionizing range. EMF pollution levels tend to be a function of line of sight, distance from emitting sources, and geographical structure of coverage area [10]. The continuous monitoring and evaluation of the EMF pollution level in crowded areas with a high density of electrical power signals have become of the utmost importance. This necessitated this study in which electric field (E) and magnetic flux density (B) pollution levels were measured in 30 different locations, namely; in school campuses, hospitals, industrial areas, markets, residential and Blantyre commercial and business center (CBC) over a two-year period during morning and evening hours. Thereafter, statistical computation was carried out to map up distribution levels of both electric and magnetic flux density pollution in the studied areas.

This manuscript briefly covers anthropogenic sources of EMF, possible heathy effects of EMF, and international guidelines for EMF exposure in chapters One and Two. Chapter Three focuses on the type of method of data collection. Geographical location of the study area has also been covered in this Chapter. Results of electric field and magnetic flux density pollution levels were measured in 30 different locations and has been discussed in Chapter Four. The results have been compared to the safety guidelines published by the International Commission on Non-Ionizing Radiation Protection (ICNIRP). Statistical analysis is also presented in Chapter Four. Lastly, the conclusion and recommendations are presented in Chapter Five of the manuscript.

## 2. Electromagnetic Fields and Human Health

### 2.1. Electromagnetic Fields

Electricity generates both magnetic and electric fields to our surrounding [8]. The magnitude of electric field strength and magnetic flux density depend on the voltage. Electric and magnetic fields surround high voltage powerlines, street powerlines, and all domestics appliances that are switched on [11]. Radiation is defined as the transmission of energy in the form of waves through space and time or through material medium including the radiated energy itself [12]. The force field associated with electric and magnetic radiation are in the vicinity through which the field is measurable [10]. When anthropogenic sources of EMF produce electromagnetic waves and are given off into the environment, it is called electromagnetic pollution.

### 2.2. Possible Effects of Electromagnetic Fields on Human Health

Nervous system of human body is one of the most vulnerable to EMF fields doses [13]. During low intense exposure of EMF fields, memory of new born babies suffer the most. Human immune system is less efficient under EMF field exposure [14]. Studies have shown that there are defects in immunity in humans under the influence of electric field [15]. Allergic people become more sensitive to magnetic and electric field. If these people stay next to energy transmission lines, they acquire pathological reactions such as convulsion and loss of consciousness [2]. Some of the low frequency magnetic guidelines for continuous and prolonged exposure have been summarized in a Table 1.

Continuous and prolonged exposure for several hours a day for several months or years has detrimental effects on humans. Electro-hypersensitive people may experience discomfort at very low EMF levels even at risk for level 1. Pregnant women should spend most of their time in level 1 but brief visits to levels 2 and 3 will probably do no harm. Everyone should avoid continuous prolonged exposure at levels 4. Bedrooms, schools, hospitals, libraries, and workplaces should be at level 1.

### 2.3. Childhood Leukaemia and Alternating Current (AC) Magnetic

Leukemia is the most common cancer in children, constituting about 35% of all malignant diseases. For example, in Germany, about 620 children out of 13.2 million below the age of 15 years are diagnosed with leukemia every year. This corresponds with an incidence of 4.8 leukemia case per 100,000 children per year. For children with an acute lymphocytic leukemia, which accounts for about 85% of all childhood leukemias, the prognosis is considerably better than that for children with acute myelocytic leukemia, for whom the five-year survival rate is still below 60%. Only very few children become ill with a chronic or a lymphocytic-myelocytic mixed leukemia [17].

The nine published studies on this topic were analyzed, with a focus on leukemia with the exposure of EMF. Consistency of a positive association and dose-response relationship are evident for assessment of past exposure. Among the five recent studies, relative risk estimates vary from 1.5 to 2.7 for past exposure assessment, and a significant dose-response relation was found in three studies. These studies have varied in quality, size, and in the way that they have assessed exposure, leading to conclusions that are sometimes inconsistent [18]. Three pooled analyses of case–control studies showed a 1.4- to 1.7-fold increased CL risk for extremely low-frequency EMF (ELF-EMF) exposure levels above 0.3 μT [16].

For occupation in shielded structures under the attenuated EMF, their health and pathological reactions are also affected negatively [19]. Low-level exposure to electric and magnetic fields have an influence on endocrine, genetic system, nervous activity, characteristic of encephalograms and psycho-physiological status in humans [11]. Resonant actions related to human physiological rhythms play a significant role in human body, yet their function is affected by EMF. Resonant amplification or attenuation of these rhythms, the appearance of harmonics and subharmonics including the results of cross modulation in nonlinear cell elements can produce a number of psychological side effects along unpredictable biological consequences [14,15].

### 2.4. Safety Limits

WHO recommendation of 12 July 1999 on the upper limit of electromagnetic fields is 300 GHz for the general public [20]. This restriction and reference level was based on the safety guidelines published by the ICNIRP [21] which was also endorsed by the Scientific Steering Committee (SSC) in the opinion on health effects of electromagnetic frequencies (EMFs) of 25 to 26 of June, 1999 [6]. Table 2 and Table 3 summaries the ICNIRP and IEEE guidelines form electric and magnetic field exposure for both general public and occupation. Malawi as a country is yet to establish its own safety requirements as regards to maximum exposure of EMF for occupation and general public arising from physical agents.

It is recommended that no permanent occupied work station or equivalent should be located in any area within one meter of the location where the magnetic flux density exceeds 50 mG (5 µT).

After more public concerns over arising health effects from the exposure of electric and magnetic flux density pollution, in order to protect the general public, the WHO established an international electric radiation project in 1996 with a goal of assessing scientific evidence of possible health effect of such radiation in the frequency range from 0–300 GHz [20]. This electric and magnetic flux density project encourages scientifically focused research to address gaps in knowledge and guide the formulation of local and internationally acceptable guidelines on upper limit of electric field radiation.

### 2.5. Precautions to Reducing the Risk of Excessive Exposure to Electric and Magnetic Flux Density Exposure

Like any type of radiation, the three perspectives of radiation protection exposure should be applied to both electric and magnetic frequency. These three are: distance from high voltage lines, time of exposure, and shielding. The electric and magnetic frequency exposure decreases with the square of distance from the pollution source. Distance protection is a non-unit system of protection, which measures the impedance between the relay location and the point where local people are and compares it with the set exposure value. The time of exposure should be kept as low as possible in carrying out a particular task for occupation exposure.

## 3. Material and Methods

This work aimed at observing the short-term electric field and magnetic flux density pollution levels in frequency band of electromagnetic exposure in school campuses, hospitals, industrial areas, markets, residential areas, and Blantyre CBC in Malawi. The measurements of electric field and magnetic flux density pollution of low and high radiofrequency origin electromagnetic waves broadcast in 30 different locations, with 2000 m intervals with highest population clusters, throughout the city of Blantyre in Malawi were performed. Trifield EMF meter model TF2 Handheld Spectrum Analyzer was used to obtain the measurements. Each measurement at a location lasted for 360 sec (6 min), which is standard measurement set by ICNIRP for a scientific meaningful result [22]. Maximum electric field and magnetic flux density were recorded with a repetition of three times and then the average value was computed. The measurements of the electric field and magnetic flux density pollution were carried out twice in a day between 10:00 and 12:00 h and 17:00 and 19:00 h. There is more power consumption during the hours of 10:00–12:00 and minimum electric power consumption between 17:00 and 19:00 h in Malawi. It was expected that mobility of people is high and most electrical devices are on during these hours.

It the data analysis, we used the Wilcoxon Signed Rank test to see if there were any significant differences in the median values of two related paired samples. This is a nonparametric test that compared the sum of ranks of absolute differences between paired observations to a critical value, also known as the *p*-value. Furthermore, we used the Kruskal Wallis test, a nonparametric test that compares the sum of ranks of the observations in each group, to find out the critical value or *p*-value, to see if there were significant differences in the median values of three or more independent groups based on ordinal or continuous data. This test was used to compare the efficacy of five different treatment groups on our outcome variable.

**Figure 1:**
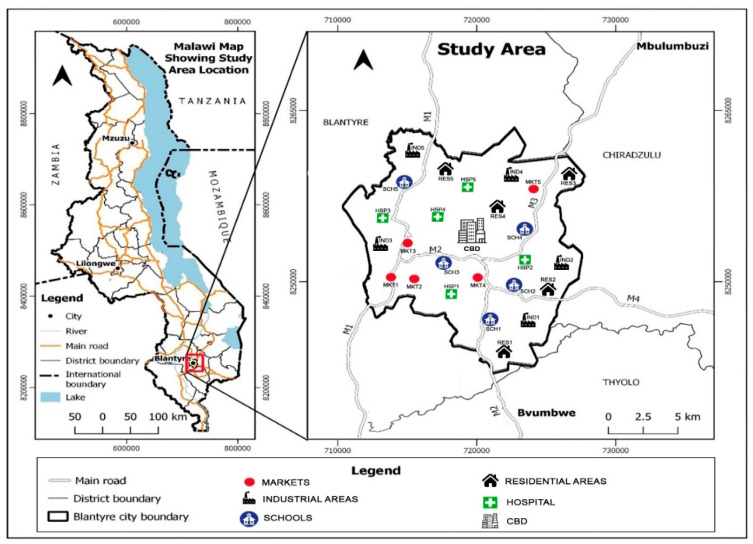
Map of measurement locations in Blantyre, Malawi.

**Figure 2:**
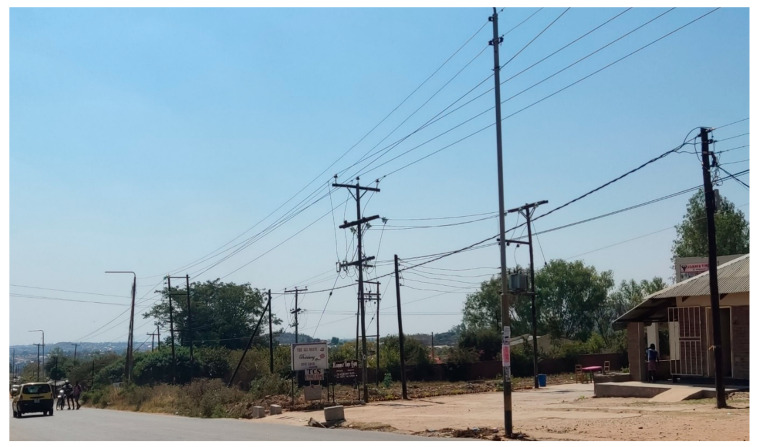
Technogenic sources of EMF pollution in one of the monitored residential areas.

The map of measurement locations selected in Blantyre City has been presented in Figure 1 and Figure 2, showing some technogenic sources of EMF pollution in one of the residential monitored areas.

## 4. Results and Analysis

The recorded maximum values of electric field (*E_Max_*) and magnetic flux density (*B_Max_*) of the statistical analysis are presented in Table 4, Table 5, Table 6, Table 7, Table 8 and Table 9. From the results, it was shown that the *E_max_* and *B_max_* values in all monitored locations for morning and evening hours were smaller than the public upper exposure limits as defined by ICNIRP of 4.2 kV/m and 2 G respectively. Nevertheless, the fact that recorded values were below the ICNIRP and WHO maximum electric field and magnetic flux density exposure limit values, does not imply that this is totally harmless. Furthermore, cumulative exposure time is much more important as long as intensity of the pollution exposure in concerned.

As shown in Table 4 and Table 6, the maximum values of recorded electric field at SCH 1, SCH 4, HSP 4, and IND 5 locations were higher, which also corresponded to higher magnetic flux density. This may be due to the fact that these locations are closer to electric base stations and that some intensive radiative devices were on at the time of monitoring. The average measured value of *E* between 10:00 and 12:00 local time in Blantyre was 249.24 mV/m, and between 17:00 and 19:00 local time was 207.85 mV/m. Similarly, maximum short-range results for *B* were 0.073 G between 10:00 and 12:00 and 0.057 G between 17:00 and 19:00 which was below the public limits of 2 G for public exposure.

From the monitored results, schools and hospitals showed that for both electric and magnetic pollution, measurements taken between 10:00 h and 12:00 h were generally higher compared to those taken between 17:00 h and 19:00 h. However, the measurements taken at industrial areas, market places, residential areas, and Blantyre CBD suggest slight differences between 10:00 h and 12:00 h and 17:00 h and 19:00 h for both electric and magnetic pollution. To determine whether there was a significant difference in electric and magnetic measurements within and across these locations, we used Wilcoxon Signed Rank test and Kruskal Wallis test. It was observed that there was no significant difference between the electric and magnetic measurements taken from 10:00 h to 12:00 h and 17:00 h to 19:00 h for schools, hospitals, industries, markets, residential, and Blantyre CBD (*p*-values > 0.05, Table 10).

The comparison of the electric field and magnetic flux density measurements across the locations was carried out to see if a particular location provided different measurements compared to the other locations. The results reveal that the electric field experienced by all the six locations was the same, that is, hospitals, schools, industries, markets, residential areas, and Blantyre CBC receive the same effect (*p* = 0.243, Table 11) for measurements carried out between 10:00 h and 12:00 h. For magnetic flux density in all the six cluster locations, the effect was also the same (*p* = 0.257, Table 12). There is no significant difference in measurements for both electric and magnetic flux density carried out from 17:00 h to 19:00 h among the six locations. Hospitals, schools, industries markets, residential, and Blantyre CBD receive the same effect between 17:00 h and 19:00 h (*p* = 0.700, Table 11 and *p* = 0.632, Table 12).

## 5. Conclusions

This study focused on electric and magnetic pollution measurements in the city of Blantyre, Malawi. The levels of electric and magnetic pollution were compared with the upper safe limits determined by ICNIRP, IEEE, and WHO for public exposure. It was observed that there were no significant differences between the electric and magnetic measurements taken from 10:00 h to 12:00 h and 17:00 h to 19:00 h for schools, hospitals, industries, markets, residential, and Blantyre CBD (*p*-values > 0.05). The average measured value of *E* between 10:00 and 12:00 local time in Blantyre was 249.24 mV/m, and between 17:00 and 19:00 local time was 207.85 mV/m. Similarly, maximum short-range results for H were 0.073 G between 10:00 and 12:00 and 0.057 G between 17:00 and 19:00, which was below the public limits of 2 G for public exposure. However, cumulative doses of both electric and magnetic pollution exposure still remain a threat to the people of Blantyre. Although the measured electric and magnetic values were lower than the exposure limits defined by international institutions, it is imperative to minimize the duration of EMF exposures to the citizenry as well as the intensity of EMF exposures to radiation as this leads to dynamic cumulative doses. Most importantly, background measurements from this study establish a baseline for future changes to be compared against.

This study proposes a standard novel mathematical empirical model for the characterization of the total electric and magnetic pollution. Additionally, a long-term measurement study of electric and magnetic pollution in different frequency bands should be conducted. Furthermore, the community awareness and participation programs (CAPPs) about health risks that could originate from exposure of electric and magnetic waves should be encouraged in Malawi. The country may adopt current electric and magnetic exposure guidelines ICNIRP 2010 to protect the public.

## Figures and Tables

**Table 1:** Low frequency magnetic guidelines for continuous and prolonged exposure [16].

Risk Level	Milligauss	Description
1	0.0–1.0	Low risk of health effects, except for electro hypersensitive people
2	1.0–2.0	Slight risk of serious health effects, mostly for children and pregnant women
3	2.0–5.0	Moderate risk of serious health effects
4	Over 5.0	High risk of serious health effects

**Table 2:** ICNIRP guidelines for EMF exposure.

Exposure (60 Hz)	Electric Field	Magnetic Field
Occupational	8.3 kV/m	1 mT (10,000 mG) *
General Public	4.2 kV/m	200 µT (2000 mG)

* 1 mG (milliGauss) = 100 nT (nanoTesla).

**Table 3:** ICNIRP and IEEE electromagnetic exposure recommendation.

	IEEE 2002	ICNIRP 2010	IEEE 2002	ICNIRP 2010
	General Public		Occupation	
Exposure general	Not specified	2000 mG	Not specified	10,000 mG
Exposure to head and torso	9040 mG	Not specified	27,100 mG	Not specified

**Table 4:** Statistical results of *E* and *B* in school campuses.

Measuring Location	Coordinates (Lat and Long)	*E* Measurements between 10:00–12:00 h	*E* Measurements between 17:00–19:00 h	ICNIRP *E* Limit Value for 6 min (Public)	*B* Measurements between 10:00–12:00 h	*B* Measurements between 17:00–19:00 h	ICNIRP *B* Limit Value for 6 min (Public)
		Max (V/m)	Max (V/m)		Max (G)	Max (G)	
SCH 1	150°47′18″ S 350°01′47″ E	4.43	5.63	4.2 kV/m	0.056	0.054	2 G
SCH 2	150°47′48″ S 350°01′22″ E	3.65	4.76		0.061	0.047	
SCH 3	150°48′42″ S 350°00′01″ E	2.75	22		0.038	0.055	
SCH 4	150°46′31″ S 350°01′37″ E	5.34	5.03		0.063	0.038	
SCH 5	150°47′18″ S 350°01′47″ E	4.75	3.64		0.75	0.029	

Note: SCH = School.

**Table 5:** Statistical results of *E* and *B* in hospitals.

Measuring Location	Coordinates (Lat and Long)	*E* Measurements between 10:00–12:00 h	*E* Measurements between 17:00–19:00 h	ICNIRP *E* Limit Value for 6 min (Public)	*B* Measurements between 10:00–12:00 h	*B* Measurements between 17:00–19:00 h	ICNIRP *B* Limit Value for 6 min (Public)
		Max (V/m)	Max (V/m)		Max (G)	Max (G)	
HSP 1	150°48′11″ S 350°01′17″ E	4.02	4.64	4.2 kV/m	0.048	0.056	2 G
HSP 2	150°35′03″ S 350°01′48″ E	4.64	4.75		0.047	0.041	
HSP 3	150°46′20″ S 350°01′28″ E	3.75	4.72		0.033	0.043	
HSP 4	150°41′35″ S 350°01′29″ E	5.04	5.03		0.067	0.037	
HSP 5	150°43′44″ S 350°00′04″ E	4.75	5.24		0.056	0.034	

Note: HSP = Hospital.

**Table 6:** Statistical results of *E* and *B* in industrial areas.

Measuring Location	Coordinates (Lat and Long)	*E* Measurements between 10:00–12:00 h	*E* Measurements between 17:00–19:00 h	ICNIRP *E* Limit Value for 6 min (Public)	*B* Measurements between 10:00–12:00 h	*B* Measurements between 17:00–19:00 h	ICNIRP *B* Limit Value for 6 min (Public)
		Max (V/m).	Max (V/m).		Max (G)	Max (G)	
IND 1	150°47′17″ S 350°01′54″ E	3.74	4.64	4.2 kV/m	0.061	0.054	2 G
IND 2	150°47′32″ S 350°01′04″ E	4.84	5.74		0.059	0.057	
IND 3	150°49′33″ S 350°00′56″ E	4.66	3.74		0.073	0.048	
IND 4	150°46′42″ S 350°01′52″ E	4.85	4.75		0.068	0.043	
IND 5	150°47′36″ S 350°58′05″ E	5.76	26		0.045	0.041	

Note: IND = Industrial area.

**Table 7:** Statistical results of *E* and *B* in markets.

Measuring Location	Coordinates (Lat and Long)	*E* Measurements between 10:00–12:00 h	*E* Measurements between 17:00–19:00 h	ICNIRP *E* Limit Value for 6 min (Public)	*B* Measurements between 10:00–12:00 h	*B* Measurements between 17:00–19:00 h	ICNIRP *B* Limit Value for 6 min (Public)
		Max (V/m).	Max (V/m).		Max (G)	Max (G)	
MKT 1	150°49′05″ S 350°01′22″ E	3.58	2.43	4.2 kV/m	0.045	0.038	2 G
MKT 2	150°48′12″ S 340°59′32″ E	2.53	3.56		0.037	0.041	
MKT 3	150°43′23″ S 350°59′47″ E	2.64	24		0.053	0.026	
MKT 4	150°46′10″ S 350°01′42″ E	3.53	2.63		0.027	0.018	
MKT 5	150°47′31″ S 350°01′19″ E	2.53	2.53		0.029	0.025	

Note: MKT = Market area.

**Table 8:** Statistical results of *E* and *B* in residential areas.

Measuring Location	Coordinates (Lat and Long)	*E* Measurements between 10:00–12:00 h	*E* Measurements between 17:00–19:00 h	ICNIRP *E* Limit Value for 6 min (Public)	*B* Measurements between 10:00–12:00 h	*B* Measurements between 17:00–19:00 h	ICNIRP *B* Limit Value for 6 min (Public)
		Max (V/m).	Max (V/m).		Max (G)	Max (G)	
RES 1	150°46′20″ S 350°01′08″ E	3.63	4.04	4.2 kV/m	0.035	0.037	8.3 kV/m
RES 2	150°47′25″ S 350°01′53″ E	2.57	3.94		0.042	0.023	
RES 3	150°48′13″ S 350°01′27″ E	3.84	3.53		0.038	0.036	
RES 4	150°49′01″ S 350°00′03″ E	2.94	2.64		0.036	0.049	
RES 5	150°47′31″ S 350°01′26″ E	3.48	3.67		0.024	0.013	

Note: RES = Residential area.

**Table 9:** Statistical results of *E* and *B* Blantyre CBC.

Measuring Location	Coordinates (Lat and Long)	*E* Measurements between 10:00–12:00 h	*E* Measurements between 17:00–19:00 h	ICNIRP *E* Limit Value for 6 min (Public)	*B* Measurements between 10:00–12:00 h	*B* Measurements between 17:00–19:00 h	ICNIRP *B* Limit Value for 6 min (Public)
		Max (V/m).	Max (V/m).		Max (G)	Max (G)	
CBC 1	150°47′03″ S 350°00′16″ E	2.47	2.54	4. 2 kV/m	0.042	0.038	2 G
CBC 2	150°47′07″ S 350°00′25″ E	3.68	2.53		0.038	0.046	
CBC 3	150°47′13″ S 350°00′41″ E	2.57	2.46		0.045	0.025	
CBC 4	150°47′18″ S 350°00′20″ E	3.64	1.45		0.027	0.027	
CBC 5	150°47′12″ S 350°00′11″ E	2.94	3.53		0.033	0.023	

Note: CBC = Central Business Centre.

**Table 10:** Difference in measurements within locations (Wilcoxon Signed Rank Test).

Location	*p*-Value (*E*)	*p*-Value (*H*)
Schools	0.50	0.45
Hospitals	0.08	0.12
Industries	0.89	0.54
Markets	0.89	0.46
Residential	0.36	0.57
Blantyre CBD	0.67	0.42

**Table 11:** Difference in electric field measurements across locations (Kruskal Wallis Test).

	10:00–12:00 h	17:00–19:00 h
Chi-Square	6.714	3.000
*p*-value	0.243	0.700

**Table 12:** Difference in magnetic flux density measurements across locations (Kruskal Wallis Test).

	10:00–12:00 h	17:00–19:00 h
Chi-Square	6.684	3.000
*p*-value	0.257	0.632

## Data Availability

Data used to support the findings of the study are available from the corresponding author upon request.
